# The “Healthcare Workers’ Wellbeing [Benessere Operatori]” Project: A Longitudinal Evaluation of Psychological Responses of Italian Healthcare Workers during the COVID-19 Pandemic

**DOI:** 10.3390/jcm11092317

**Published:** 2022-04-21

**Authors:** Gaia Perego, Federica Cugnata, Chiara Brombin, Francesca Milano, Emanuele Preti, Rossella Di Pierro, Chiara De Panfilis, Fabio Madeddu, Valentina Elisabetta Di Mattei

**Affiliations:** 1 Department of Psychology, University of Milano-Bicocca, 20126 Milan, Italy; emanuele.preti@unimib.it (E.P.); rossella.dipierro@unimib.it (R.D.P.); fabio.madeddu@unimib.it (F.M.); 2 School of Psychology, Vita-Salute San Raffaele University, 20132 Milan, Italy; cugnata.federica@hsr.it (F.C.); brombin.chiara@hsr.it (C.B.); dimattei.valentina@hsr.it (V.E.D.M.); 3 University Centre for Statistics in the Biomedical Sciences (CUSSB), Vita-Salute San Raffaele University, 20132 Milan, Italy; 4 Clinical and Health Psychology Unit, IRCCS San Raffaele Scientific Institute, 20132 Milan, Italy; milano.francesca96@gmail.com; 5 Department of Medicine and Surgery, University of Parma, 43121 Parma, Italy; chiara.depanfilis@unipr.it

**Keywords:** COVID-19, healthcare workers, mental health, mixed effects model, Random Effects/Expectation Maximization (RE-EM) Tree

## Abstract

Background: COVID-19 forced healthcare workers to work in unprecedented and critical circumstances, exacerbating already-problematic and stressful working conditions. The “Healthcare workers’ wellbeing (Benessere Operatori)” project aimed at identifying psychological and personal factors, influencing individuals’ responses to the COVID-19 pandemic. Methods: 291 healthcare workers took part in the project by answering an online questionnaire twice (after the first wave of COVID-19 and during the second wave) and completing questions on socio-demographic and work-related information, the Depression Anxiety Stress Scale-21, the Insomnia Severity Index, the Impact of Event Scale-Revised, the State-Trait Anger Expression Inventory-2, the Maslach Burnout Inventory, the Multidimensional Scale of Perceived Social Support, and the Brief Cope. Results: Higher levels of worry, worse working conditions, a previous history of psychiatric illness, being a nurse, older age, and avoidant and emotion-focused coping strategies seem to be risk factors for healthcare workers’ mental health. High levels of perceived social support, the attendance of emergency training, and problem-focused coping strategies play a protective role. Conclusions: An innovative, and more flexible, data mining statistical approach (i.e., a regression trees approach for repeated measures data) allowed us to identify risk factors and derive classification rules that could be helpful to implement targeted interventions for healthcare workers.

## 1. Introduction

Healthcare settings may represent a challenging workplace, characterized by long and undefined working hours, excessive workloads, competitiveness of training, high responsibility, and constant exposure to suffering, illness, and mourning [1,2]. However, lack of time, stigma, and concerns around confidentiality may prevent seeking psychological support [2,3].

Consistently, increasing evidence shows that healthcare workers around the world report high levels of depression, anxiety, stress, burnout, and post-traumatic stress disorder (PTSD) [1,4]. However, the literature is limited, and the samples analyzed are heterogeneous, resulting in a prevalence of psychiatric symptoms ranging from 30% to 60% for physicians [5,6,7] and from 11% to 73% for nurses [8]. 

In turn, neglected mental health issues in healthcare workers can affect both team and individual work performance, resulting in a reduced quality of care [9], lower patient satisfaction, and higher rates of medical errors and staff turnover [1,9,10], with a remarkable impact on the healthcare economy [1,10,11].

This topic is currently of critical importance, given the detrimental consequences of the COVID-19 pandemic on the entire population, e.g., ref. [12] and healthcare workers in particular [13], as shown in previous epidemics [14], worsening already-problematic and stressful workplace conditions. Indeed, frontline healthcare workers have been working for more than a year in unprecedented and critical circumstances to cope with the COVID-19 pandemic, while being exposed to potentially traumatic or stressful factors such as fear of contagion, a lack of personal protection equipment, longer working hours, countless patient deaths and numerous critical patients, and continuous updates to hospital procedures [10,15].

Although healthcare workers faced the second wave of the pandemic with more therapeutic knowledge than the first, they still had limited resources to care for COVID-19 patients. In fact, the unpredictability of the disease and the pandemic’s course, the extremely high number of deaths and critical patients, and the necessity to make difficult choices about prioritizing care remained serious concerns about COVID-19 [15].

Several reviews and meta-analyses show that working in COVID-19 wards affected healthcare workers’ mental health in terms of high rates of depression, anxiety, insomnia, burnout, and PTSD symptoms [13,15,16,17,18]. A recent meta-analysis [10] found the following pooled prevalence of psychiatric outcomes among healthcare workers: 30% for anxiety, 31.1% for depression, 56.5% for acute stress, 20.2% for post-traumatic stress, and 44% for sleep disorders. However, as other two meta-analyses pointed out [19,20], most healthcare workers actually experienced mild psychiatric symptoms, with moderate and severe symptoms being less common.

This is in line with our baseline findings, which point to low or mild mental health issues among healthcare workers after the main peak of the outbreak’s first phase [21]. Notwithstanding, the pandemic has evolved quickly, and early studies were unable to capture post-traumatic stress disorders and the mental health outcomes associated with a state of prolonged stress. Longitudinal studies are, thus, required to analyze the effect of time on these psychiatric outcomes [10] and to differentiate the effect of the pandemic from all other pre-existing stressors in the hospital work environment [22].

In this contribution, we present findings of the “Healthcare workers’ wellbeing [Benessere Operatori]” project, which aims at evaluating psychological distress, as well as socio-demographic, situational, and personal factors that may affect individuals’ psychological responses to the COVID-19 pandemic. All these factors have been assessed twice: at baseline (between 9 May and 13 July 2020, after the main peak of the COVID-19 outbreak in Italy) and during the second wave (between 5 and 30 December 2020). Along with more traditional mixed effects modelling approaches, an alternative advanced data mining approach, extending regression trees methodology to repeated measures data, has been applied. The applied procedure is extremely flexible and appealing since it allows for the identification of the best variables with the best cut-off values for discriminating among different outcome responses, while uncovering complex relationships among predictors. It thus provides an effective tool to be used in clinical practice to support decision making process.

## 2. Materials and Methods

### 2.1. Participants and Procedure

The study was conducted according to the guidelines of the Declaration of Helsinki and approved by the Ethics Committee of the University of Milano-Bicocca (protocol n. 0024531/20), the Ethics Committee of the IRCCS San Raffaele Scientific Institute (protocol n. 109/2020), and the Ethics Committee of the Parma Local Health Authority (protocol n. PG0019826_2020).

This study is part of a web-based longitudinal project to examine the psychological impact of COVID-19 on a sample of Italian healthcare workers involved in the management of the pandemic. After reading the informed consent, participants voluntarily completed an online survey, administered through Qualtrics and sent to the e-mail address provided, during the baseline assessment. We assessed participants’ working conditions, individual perception of the COVID-19 situation, anxiety, depression, and insomnia symptoms, post-traumatic stress, state anger, and burnout levels. In the present study, we will also analyze the coping strategies and perceived social support measured during the baseline study.

In total, 344 healthcare workers participated in the second survey, ten of whom stated that they had not worked in the previous three months and were thus excluded from the analysis. Finally, statistical analyses have been carried out on a sample of 291 respondents with complete records on both demographic and psychological variables.

### 2.2. Measures

A self-report questionnaire was used to collect socio-demographic and work-related information from participants, including their age, gender, psychological/psychiatric history, ward in which they worked, and whether they had received emergency training.

The Depression Anxiety Stress Scale (DASS-21) [23,24] is a 21-item scale that assesses general distress using a tripartite model of psychopathology. This questionnaire includes three subscales: depression, anxiety, and stress. Each item is rated on a four-level Likert scale (0 = never; 3 = almost always). The total score is calculated by adding together the response values for each item. Higher scores suggest severe levels of depressive, anxiety, and stress symptoms. The original version of the questionnaire showed an internal reliability with Cronbach’s alpha coefficient of 0.91 for the depression scale, 0.84 for the anxiety scale, and 0.90 for the stress scale [24]. The total score of the Italian version reported a Cronbach’s alpha value of 0.90, with subscale values ranging from 0.74 to 0.85 [23].

The Insomnia Severity Index (ISI) [25,26] is a self-report questionnaire that assesses the nature, severity, and impact of insomnia using seven items rated on a five-level Likert scale (0 = “no problem”; 4 = “very severe problem”), with scores ranging from 0 to 28. The dimensions evaluated are severity of sleep onset, sleep maintenance, early morning awakening problems, sleep dissatisfaction, interference of sleep difficulties with daytime functioning, noticeability of sleep problems by others, and distress caused by the sleep difficulties. The original version of the ISI reported a Cronbach’s alpha coefficient of 0.74 [26]. The Italian version showed a Cronbach’s alpha coefficient of 0.75 [25].

The Impact of Event Scale-Revised (IES-R) [27,28] is a 22-item self-report questionnaire for evaluating the frequency of intrusive and avoidant thoughts and behaviors associated with a traumatic event. Items are rated on a five-point Likert scale (0 = “not at all”; 4 = “extremely”). The IES-R is divided into three subscales. Intrusion (8 items) assesses intrusive thoughts, nightmares, intrusive feelings, and imagery related to the traumatic event; Avoidance (8 items) evaluates avoidance of feelings, situations, and ideas; Hyperarousal (6 items) measures difficulty in concentrating, anger and irritability, psychophysiological arousal in response to reminders, and hypervigilance. The original version showed high levels of internal consistency (Intrusion: α = 0.87–0.94, Avoidance: α = 0.84–0.87, Hyperarousal: α = 0.79–0.91) [28]. The Italian version shows good (0.84) and acceptable (0.71) internal consistency for the intrusion subscale and the avoidance subscale, respectively [27].

The State-Trait Anger Expression Inventory-2 (STAXI-2) [29] is a 57-item self-report questionnaire that measures five domains of anger: State-Anger, Trait-Anger, Anger Expression-In, Anger Expression-Out, and Anger-Control. Responses are rated on a four-point Likert scale, ranging from 1 (not at all) to 4 (almost always). Cronbach’s α coefficients range from 0.73 to 0.76, indicating high internal reliability for all the subscales except for the Trait Anger Scale/Angry Reaction [29]. In the present study, we only used the State-Anger subscale to assess healthcare workers’ acute reaction to the pandemic.

The Maslach Burnout Inventory (MBI) [30,31] consists of 22 items, divided into three subscales, that assess the three components of the burnout syndrome: emotional exhaustion (9 items), depersonalization (5 items), and professional realization (8 items). Each item is rated on a seven-point Likert scale (0 = “never”; 6 = “every day”). The subscales showed good internal consistency both for the original version (α = 0.71–0.90) [30] and for the Italian version (α = 0.68–0.87) [31].

The Brief Cope [32,33] is a 28-item questionnaire, divided into 14 subscales, measuring coping responses. Each item is rated on a four-level Likert scale (0 = “I have not been doing this at all”; 3 = “I have been doing this a lot”). Coping strategies can be grouped into problem-focused (strategies aimed at changing a stressful situation: active coping, use of instrumental support, positive reframing, and planning), emotion-focused (strategies to regulate emotions associated with a stressful situation: use of emotional support, venting, humor, acceptance, self-blame, religion), and avoidance coping strategies (physical or cognitive efforts to disengage from the stressor: self-distraction, denial, substance use, behavioral disengagement) [34,35]. The original version of the questionnaire showed Cronbach’s alpha coefficients, ranging from 0.50 to 0.90 [32], while the Italian version of the questionnaire revealed omega coefficients for reliability, ranging from 0.439 to 0.959 [33]. 

The Multidimensional Scale of Perceived Social Support (MSPSS) [36,37] is a 12-item self-administered questionnaire evaluating social support perceived by family, friends, and significant others, rated on a 7-point Likert scale (1 = “very strongly disagree”; 7 = “very strongly agree”). Higher scores indicate higher perceived social support. The internal reliability of the questionnaire is good, with Cronbach’s alpha coefficients ranging from 0.85 to 0.91 [38]. The Italian version shows good indices of reliability with Cronbach’s alpha coefficients ranging from 0.81 to 0.98 [36]. 

Furthermore, we assessed how worried participants were about the possibility that themselves, their relatives, their friends, and their colleagues could contract COVID-19. Four items were rated on a five-point Likert scale (1 = “not at all”; 5 = “extremely”). A total score of worry was obtained by averaging items scores.

Finally, we evaluated participants’ working conditions over the previous three months in several areas, including eating, sleeping, working shifts, isolation, and wearing appropriate protective equipment. Seven items were rated on a five-point Likert scale (1 = “not at all”; 5 = “very much”). A total score of working conditions was obtained by averaging item scores. Higher scores indicate worse working conditions.

### 2.3. Statistical Analysis 

Median and interquartile range (IQR) were used as summary statistics to describe continuous variables, while categorical variables were expressed as frequencies and percentages. Radar plots were used to visualize differences between measurements collected during the two evaluations in the different healthcare worker categories.

Linear mixed-effects (LME) models [39] were applied to evaluate the changes in the psychological outcomes over time while accounting for respondent-specific heterogeneity through random effects specification. The variables included in the models were: time (categorical with two levels, T0 and T1, respectively, at baseline and during the second wave), gender, occupation, working or having worked in COVID-19 wards (time dependent variable), worry scores and the evaluation of working conditions, the presence of psychological or psychiatric symptoms in the past, having attended emergency training, perceived social support as measured by the MSPSS, and the three Brief COPE subscales. To highlight specific differences in the outcome variables over time for the different healthcare workers categories, we also entered in the model the interaction between occupation and time.

Standard transformations (square root, power, ordered quantile normalization) were applied to outcome variables to satisfy model regression assumptions.

To examine psychological measure dynamics over time, within a data mining framework, an extension of regression trees methodology accounting for correlation structure among observations was considered. This approach is suited for studies with repeated measures and longitudinal data. The tree-based estimation method, proposed and implemented in the R RE-EM tree package by Sela and Simonoff [40], was considered.

A regression tree [41] implements a binary recursive partitioning in which predictor variables that best discriminate among response profiles, along with the optimal cut-points, are automatically chosen. The splitting criterion is based on maximizing the reduction in the sum of squares, for the node, until convergence is reached. The approach is very flexible in handling missing values and both quantitative and qualitative predictors. 

Following the branches of the resulting tree, hence considering rules derived from the variables’ best cut-off values, it is possible to derive different patterns of longitudinal responses. Regression trees provide an effective tool for supporting decision-making in clinical practice and identifying relevant variables associated with different outcomes, while uncovering complex relationships among predictors. Trees were estimated using the same covariates included in the mixed models. All the analyses were performed using R statistical software (version 4.1.1, https://cran.r-project.org/index.html, accessed on 10 October 2021). The significance level was set at 0.05.

## 3. Results

Participants’ characteristics are shown in Table 1. The final sample included 291 participants. The median age was 46 years (IQR = [35.00, 54.00]), ranging from 23 to 72 years; 239 (82.1%) were female.

Among the sample, 23% reported having a psychological/psychiatric history, and only 16.8% reported having undergone emergency training.

Concerning their occupation, 31.3% of the participants were physicians, 33.3% were nurses, 27.9% were other healthcare workers, and 7.6% were clerks. At T0, 33.3% of the participants worked in a COVID-19 ward, and 15.5% worked in a COVID-19 ward at T1. 

**Figure 1:**
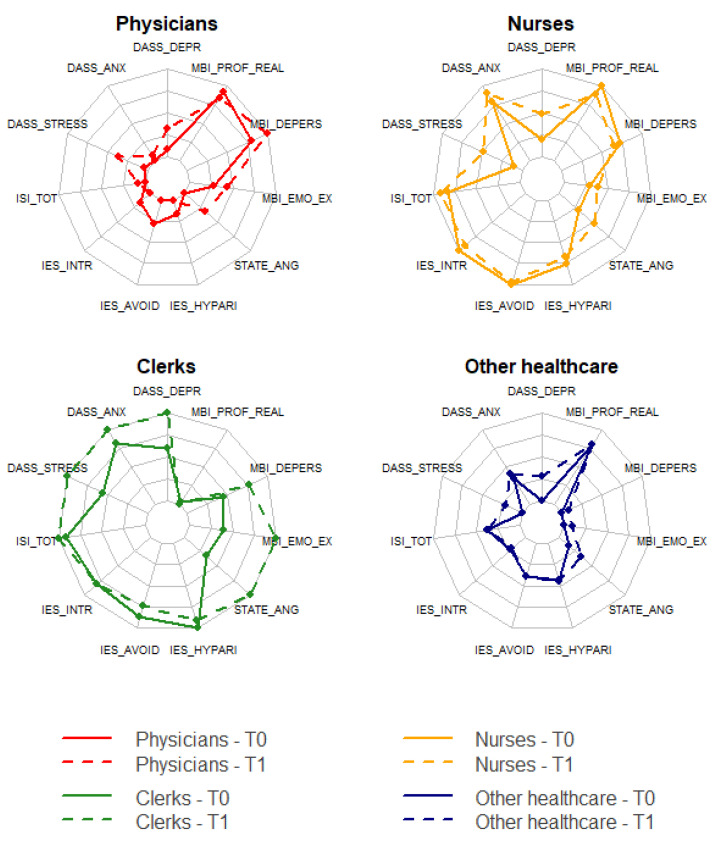
Radar charts displaying average scores of the investigated psychometric variables at T0 and T1, stratified by occupation (Table S1). Out of the 291 participants, 91 were physicians, 97 were nurses, 81 were other healthcare workers, and 22 were clerks. Abbreviation: DASS Depr, Depression Anxiety Stress Scale-Depression; DASS Anx, Depression Anxiety Stress Scale-Anxiety; DASS Stress, Depression Anxiety Stress Scale-Stress; MBI Prof Real, Maslach Burnout Inventory-Professional Realization; MBI Depers, Maslach Burnout Inventory-Depersonalization; MBI Emo Ex, Maslach Burnout Inventory-Emotional Exhaustion; STATE Ang, STATE Anger; IES Hypar, Impact of Event Scale- Hyperarousal; IES Avoid, Impact of Event Scale-Avoidance; IES Intr, Impact of Event Scale-Intrusion; ISI Tot, Insomnia Severity Index Total score.

Table 2 shows the characteristics of the participants stratified by occupation, and radar plots in Figure 1 display the average score of the psychological constructs of interest for each occupation group at each time point. Overall clerks reported higher average scores on almost all the scales, and their psychological condition seems to worsen, at least at a descriptive level, at the second time point. State-anger, DASS-21 scales, and emotional exhaustion subscale are, on average, higher for all the healthcare workers at the second time point (complete descriptive statistics are reported in Table S1). Observed ranges for all the psychometric scales are reported in Table S2.

### 3.1. DASS-21—Depression, Anxiety, and Stress

Table 3 displays the estimated models for the DASS-21 subscales. DASS-21 subscales are not significantly different at T1, with respect to the baseline values (T0). Higher levels of worry, worse working conditions, and having a psychological/psychiatric history significantly increase anxiety, depression, and stress levels. Having undergone emergency training significantly decreases all DASS-21 subscales. Higher levels of perceived social support decrease only participants’ depression levels. Considering the effects of coping strategies, we found that the use of problem-focused coping significantly decreases all DASS-21 subscales, avoidant coping significantly increases all DASS-21 subscales, and emotional-focused coping significantly increases only depression and stress levels. Finally, nurses show higher anxiety levels than physicians. 

**Figure 2:**
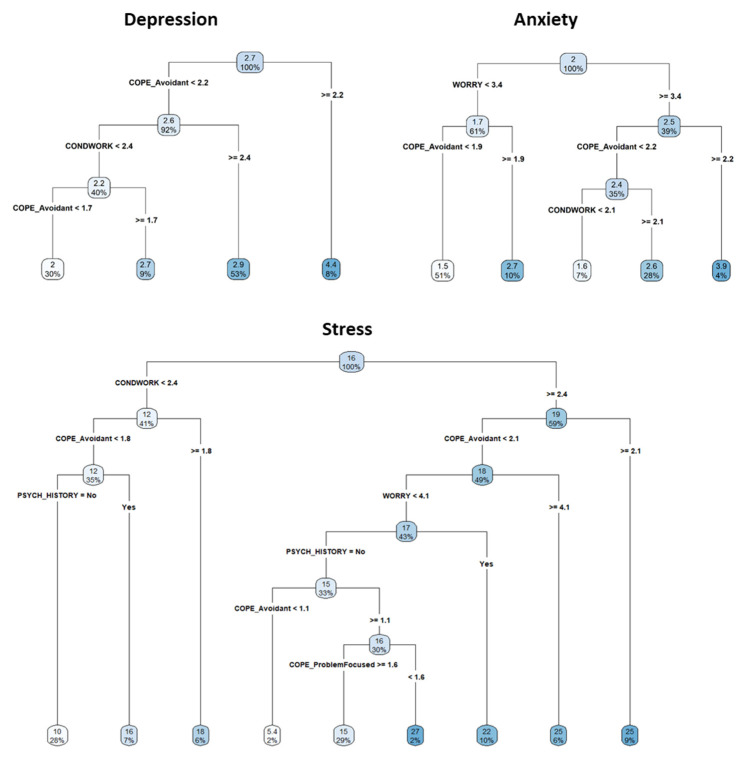
RE-EM tree for the DASS-21 subscales. Depression and anxiety scales were square root transformed. Each tree represents a series of splits starting at the top of the tree. Starting from the top node, a series of questions are presented based on the splitting variables and corresponding cut-off values. Depending on the answer, other branches may appear until the final node, which displays the average predicted outcome value for participants, satisfying all the conditions, leading to that node and the proportion of subjects falling in the node itself. For example, in the first tree for the depression scale, the top split assigns observations having avoidant coping scores greater than or equal to 2.2 to the right branch. The predicted depression level for these subjects is given by the mean response value for the individuals in the data set with avoidant coping ≥ 2.2. For such subjects, the mean depression level is 4.4. Among subjects who have avoidant coping scores < 2.2, the working conditions also affect depression level. For subjects with avoidant coping score < 2.2 and working conditions ≥ 2.4, the predicted depression level is 2.9. For subjects having working conditions < 2.4 and avoidant coping scores between 1.7 and 2.2, the predicted depression level is 2.7, whereas for subjects having a working conditions score < 2.4 and avoidant coping scores lower than 1.7, the predicted depression level is 2. The same logic applies to all the other trees. Abbreviation: CONDWORK, working conditions; Psych History, psychiatric history.

Figure 2 shows the estimated regression trees for the prediction of the DASS-21 subscales scores. For depression, among all the variables, the algorithm selected both avoidant coping and working conditions as the variables best discriminating among participants’ response profiles. High levels of avoidant coping and worse working conditions predict the highest levels of depression, whereas low levels of avoidant coping and better working conditions predict lower levels of depression symptoms. For subjects with avoidant coping ≥ 2.2, the mean depression level is 4.4. For subjects having a working conditions score < 2.4 and avoidant coping scores lower than 1.7, instead, the predicted depression level is 2.

When anxiety is considered as an outcome, in addition to avoidant coping and working conditions, the algorithm selected the worry scale. 

Following the tree branches, participants with higher scores on the worry scale (≥3.4) and avoidant coping scores, greater than or equal to 2.2, show the highest average value equal to 3.9. Conversely, participants with scores on the worry scale lower than 3.4, and showing avoidant coping scores lower than 1.9, show the lowest average value equal to 1.5. In the tree for stress, psychiatric/psychological history and problem-focused coping strategy were selected, in addition to splitting variables characterizing the other two trees, to best discriminate among different response profiles.

### 3.2. MBI Emotional Exhaustion, Insomnia, IES-R Intrusion, and State Anger

Table 4 shows the estimated models for the MBI emotional exhaustion scale, the ISI score, the IES-R Intrusion score, and the State anger score. When comparing the two time points, the state anger score significantly increases at T1 with respect to the baseline values (T0). Only for clerks, the MBI emotional exhaustions score also significantly increases at T1 compared to T0. Focusing on the occupation, overall, nurses report higher ISI levels and IES-R Intrusion levels than physicians. Higher levels of worry and worse working conditions significantly increase all of the considered scales. A previous history of psychological or psychiatric symptoms significantly increases the MBI emotional exhaustion score, the ISI score, and the IES-R Intrusion score but not the state anger score. Older age significantly increases the ISI score and the IES-R Intrusion score. Having undergone emergency training significantly decreases the ISI and IES-R Intrusion scores. Higher levels of perceived social support decrease the MBI emotional exhaustion score. Finally, we found that using avoidant coping significantly increases all considered scales, and the use of problem-focused and emotion-focused coping strategies significantly influences the MBI emotional exhaustion scale and state anger scale.

**Figure 3:**
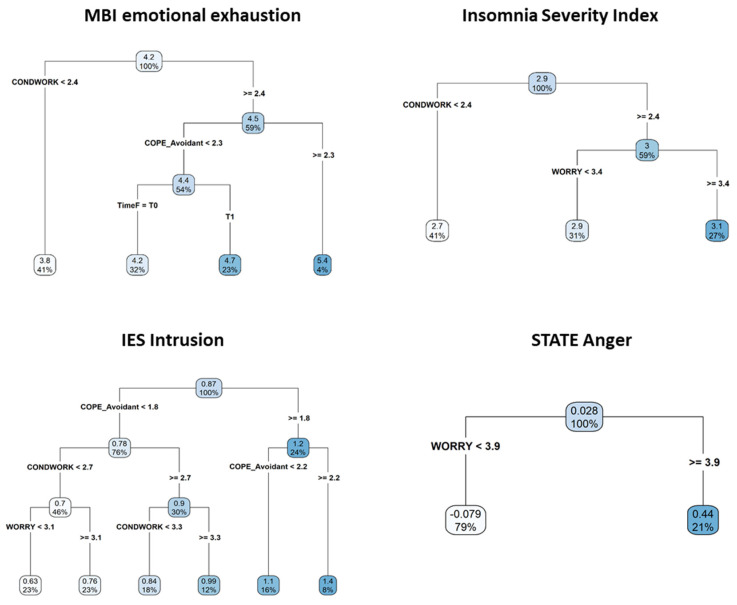
Estimated regression trees for Emotional exhaustion, Insomnia, Intrusion, and State Anger. Square root transformation was applied to the MBI Emotional Exhaustion scale, ISI total score, and IES-R Intrusion scale, while ordered quantile normalization was used for the State Anger scale. Abbreviation: CONDWORK, working conditions.

Figure 3 shows the estimated regression trees for the same outcomes.

For emotional exhaustion, the algorithm selected working conditions, avoidant coping strategies, and survey administration time as the best discriminating variables. Worse working conditions and higher use of avoidant coping strategies will lead to the highest predicted levels of emotional exhaustion, whereas the lowest outcome score is predicted for participants with better working conditions (<2.4). Moreover, worse working conditions and avoidant coping scores lower than 2.3, depending on the survey administration time, will lead to different predicted outcome scores, with higher predicted values at the time of the second survey.

For insomnia, the algorithm selected working conditions and the worry scale as the best discriminating variables. Worse working conditions and higher levels of worry will lead to the highest predicted levels of insomnia, whereas the lowest outcome score is predicted for participants with better working conditions (<2.4). Participants with worse working conditions and a level of worry lower than 3.4 will have predicted values lying in the middle and between those found in the previous two scenarios.

With reference to intrusion, working conditions, avoidant coping strategies, and scores on the worry scale are selected as the best splitting variables to identify different response profiles. Higher use of avoidant coping strategies (≥1.8) will lead to higher predicted levels of intrusion.

When avoidant coping strategies are lower than 1.8 and associated with better working conditions and lower levels of worry, the lowest levels of intrusion are predicted.

When avoidant coping strategies are lower than 1.8 and associated with worse working conditions (>2.7), participant profiles, with levels of intrusion lying in the middle, between the best and the worst scenarios, are identified. 

For state anger, out of all the variables, only the worry scale plays a role in discriminating among participants’ response profiles, with higher levels of worry leading to the highest predicted state anger score. 

Estimated LME models for depersonalization and professional realization MBI subscales are reported in Table S3. In Table S4, LME models for the IES-R subscales of avoidance and hyperarousal are reported.

## 4. Discussion

The present study is the second phase of a longitudinal study investigating the psychological consequences of the COVID-19 outbreak and their predictive factors in a sample of Italian healthcare workers. To our knowledge, this is one of the few longitudinal studies that monitored the mental health of healthcare workers during the COVID-19 outbreak [22,42,43]. 

In general, the scores obtained by our sample on the psychological scales during the second phase do not significantly differ from the scores obtained during the first phase. Overall, regardless of the category of healthcare workers, our sample only showed an increase in state anger levels. Furthermore, during the second wave, clerks seemed to experience higher levels of burnout symptoms.

These results are in line with the scant literature, showing a substantial invariance of psychiatric symptoms among healthcare workers throughout the epidemic [22,42,43].

However, working under chronic stress conditions and experiencing a poor quality of sleep both contribute to increased feelings of anger among healthcare workers during the COVID-19 outbreak [44,45]. Moreover, during the second wave of the pandemic, public opinion appears to have shifted, and healthcare workers now feel less support, appreciation, and trust from the general population, and they are viewed less favorably than in the past [22]. Consistently, during previous epidemics, feelings of anger seemed to be frequent among healthcare workers, caused by both long-term stressful working conditions and poor adherence to infection control guidelines, as well as rejection and stigma experienced from relatives and public opinion [46]. 

Furthermore, the increasing levels of burnout experienced by clerks during the second phase may be explained by the end of smart-working and the subsequent return to work in hospitals. 

Concerning predictive factors, our results highlight that higher levels of worry, worse working conditions, a previous history of psychiatric illness, being a nurse, older age, and avoidant and emotion-focused coping strategies seem to be risk factors for healthcare workers’ mental health. Conversely, high levels of perceived social support, the attendance of emergency training, and problem-focused coping strategies seem to be protective factors for healthcare workers’ mental health. 

Specifically, worse working conditions and worry about the infection represent risk factors for higher levels of depression, anxiety, stress, burnout, PTSD, insomnia, and state anger. Considering the regression trees, a score greater than or equal to 2.4 in working conditions is a cut-off for higher depression scores (for participants showing avoidant coping scores lower than 2.2), stress, burnout, and insomnia; a high worry score (≥3.9) is the only factor that distinguishes between the highest and lowest levels of state anger, whereas a score greater than or equal to 3.4 discriminates among participants with more extreme levels of anxiety and insomnia.

These findings are in line with the literature showing that difficult working environments, including poor supervision and organizational support, intense workloads, a lack of personal protective equipment or its continuous use for many hours, and fear and concern about becoming infected or infecting relatives or colleagues, increase the psychological distress’ levels of healthcare workers [47,48,49]. 

Concerning demographic variables, older age seems a risk factor for higher levels of insomnia and intrusion symptoms. This result is consistent with the literature, which shows an association between increasing age and a physiological decline in sleep quality [50]. Additionally, the literature identifies older age as a risk factor for PTSD symptoms during the COVID-19 pandemic, probably due to the elderly being a high-risk category for infection and death [51,52].

Moreover, a previous history of psychiatric illness seems to be a risk factor for higher levels of depression, anxiety, stress, burnout, PTSD, and insomnia symptoms. The current literature highlights that a history of psychiatric symptoms seems to make healthcare workers more vulnerable and more likely to experience psychological distress during the COVID-19 emergency [53,54]. 

Furthermore, being a nurse seems to be a risk factor for higher levels of anxiety, PTSD intrusion symptoms, and insomnia. This result is consistent with the literature showing that being a nurse represents a risk factor for worse mental health, which is probably due to the longer time spent with patients in contact with their fears, suffering, and death [18,49,55,56].

Concerning protective factors, having undergone emergency training predicts low levels of depression, anxiety, stress, PTSD, and insomnia symptoms. This result is in line with the literature showing perceived adequacy of training as a protective factor for long-term psychiatric morbidity [57], post-traumatic stress [58,59], and burnout [58] in previous epidemics. This is understandable given that the goal of emergency training is to improve healthcare workers’ skills and abilities to increase their sense of control and avoid being overwhelmed during a real emergency [60]. 

High levels of perceived social support from family and friends seem to be a protective factor for lower depression and burnout symptoms. In line with the literature, high perceived social support from family, colleagues, and friends is helpful to deal with work-related stress and to increase self-confidence [18,47].

Concerning coping strategies, regression trees highlighted that higher avoidant coping scores lead to the highest scores in the depression, anxiety, stress, burnout, and PTSD subscales.

The detrimental role of avoidant coping strategies is consistent with current and past epidemic literature [17,61,62,63]. Avoidance strategies, such as denial or self-distraction, may be helpful for short periods of time to allow the person to continue with their tasks while also giving them some time to think. However, in the long-term, these coping strategies are dangerous for mental health, provoking dysfunctional detachment and distance from the problem, while changing neither the situation nor the associated psychological distress [64,65]. 

Conversely, the use of problem-focused coping strategies seems to be a protective factor for lower levels of depression, anxiety, stress, burnout, and state anger. This result is in accordance with the literature, showing that these strategies can increase feelings of autonomy and self-efficacy while reducing psychological distress [17,61,66]. These coping strategies may help in changing the meaning of the event and focusing on a specific goal, thereby increasing the perception of control, and avoiding being overwhelmed by the stressful situation [64,65].

Finally, the use of emotion-focused coping strategies seems to be a risk factor for higher levels of depression, stress, burnout, and state anger. The literature concerning emotion-focused coping strategies is contradictory [17,64], which is probably due to the heterogeneous nature of the strategies included in this subscale (e.g., use of emotional support and self-blaming). Moreover, during an emergency, problem-focused strategies appear more effective and adaptive than emotion-focused strategies [67]. Making difficult decisions in a stressful and extraordinary situation, with little knowledge about the disease and poor support from family and friends due to isolation, may be overwhelming if emotions are prioritized over the problem. Indeed, because of the governments’ social restrictions policy in response to the COVID-19 emergency, a typically positive and protective coping strategy, such as the use of emotional support [66,68], could be a risk factor for poor mental health among healthcare workers [63,66,67,69].

In general, our findings align with other Mediterranean studies on the psychological impact of the COVID-19 pandemic on healthcare workers. Indeed, studies conducted in Italy, Greece, Portugal, Spain, and France highlighted a more resilient attitude among healthcare workers compared to studies conducted in China, the United Kingdom, and the Middle East [70]. Moreover, worse working conditions, higher levels of worry about the infection, and a previous history of psychiatric illness seem to be the main risk factors for poor mental health among Mediterranean healthcare workers [71,72,73,74].

### Limitations and Strengths

Among limitations to be acknowledged, a self-selection bias may have occurred, as only participants experiencing low levels of psychological distress may have taken part in this research. Then, as only a subset of participants completed the assessment twice, the study sample may not be representative of both the healthcare worker population and the initial study sample.

However, this longitudinal study allowed us to monitor the mental health of healthcare workers over time and investigate relationships among variables to identify potential target risk and protective factors. Another strength of our study is the inclusion of healthcare workers from various hospitals located throughout Italy. Finally, the use of an online survey may have encouraged participants to reveal sensitive aspects of their work without worrying about confidentiality [75].

## 5. Conclusions

Healthcare workers seemed to be resilient in the face of the pandemic. Their mental health and general wellbeing is a complex issue that the government should monitor. In the current context, much can be offered, such as virtual clinics and remotely delivered psychological therapies and psychoeducation. However, it is also necessary to reduce mental health stigma, as physicians appear generally reluctant to disclose their problems, even when they are experiencing significant psychological distress [76]. The proposed data mining approach allowed us to derive classification rules and to identify risk and protective factors for healthcare workers’ psychological wellbeing, which should be monitored during emergency situations. The proposed statistical methodology could provide more insight into the psychological aspects which are leveraged when implementing training/intervention programs.

## Figures and Tables

**Table 1:** Demographic, clinical, and occupational characteristics (*n* = 291).

**Age** (median [IQR])—years	46.00 [35.00, 54.00]
**Gender = Female**—no. (%)	239 (82.1%)
**Psych history = Yes**—no. (%)	67 (23.0%)
**Emergency training = Yes**—no. (%)	49 (16.8%)
**Occupation**	
Physicians—no. (%)	91 (31.3%)
Nurses—no. (%)	97 (33.3%)
Clerks—no. (%)	22 (7.6%)
Other healthcare—no. (%)	81 (27.8%)
**T0—Ward COVID-19 = Yes**—no. (%)	97 (33.3%)
**T1—Ward COVID-19 = Yes**—no. (%)	45 (15.5%)

Abbreviation: no., number; Psych History, psychiatric history.

**Table 2:** Demographic, clinical, and occupational characteristics stratified by occupation (*n* = 291).

	Physicians (*n* = 91)	Nurses (*n* = 97)	Clerks (*n* = 22)	Other Healthcare (*n* = 81)
**Age** (median [IQR])—years	46.00 [36.00, 56.50]	45.00 [34.00, 51.00]	46.50 [38.00, 53.00]	46.00 [34.00, 54.00]
**Gender = Female**—no. (%)	60 (65.9%)	88 (90.7%)	17 (77.3%)	74 (91.4%)
**Psych history = Yes**—no. (%)	28 (30.8%)	15 (15.5%)	4 (18.2%)	20 (24.7%)
**Emergency training = Yes**—no. (%)	16 (17.6%)	26 (26.8%)	-	7 (8.6%)
**T0—Ward COVID-19 = Yes**—no. (%)	27 (29.7%)	54 (55.7%)	-	16 (19.8%)
**T1—Ward COVID-19 = Yes**—no. (%)	13 (14.3%)	24 (24.7%)	-	8 (9.9%)
**MSPSS** (median [IQR])	72.00 [62.00, 77.00]	71.00 [61.00, 79.00]	67.00 [57.25, 75.75]	71.00 [62.00, 78.00]
**COPE—Problem-Focused** (median [IQR])	2.88 [2.50, 3.25]	3.00 [2.62, 3.38]	2.94 [2.75, 3.22]	3.00 [2.50, 3.38]
**COPE—Emotion-Focused** (median [IQR])	2.25 [2.00, 2.50]	2.33 [2.17, 2.67]	2.25 [1.88, 2.40]	2.33 [2.08, 2.58]
**COPE—Avoidant** (median [IQR])	1.38 [1.19, 1.75]	1.62 [1.38, 1.88]	1.62 [1.38, 1.97]	1.50 [1.38, 1.75]
**T0—WORRY** (median [IQR])	3.00 [2.50, 3.25]	3.25 [2.75, 3.75]	3.25 [2.81, 4.00]	3.25 [3.00, 3.75]
**T0—CONDWORK** (median [IQR])	2.71 [2.41, 3.23]	2.86 [2.29, 3.50]	2.90 [2.45, 3.55]	2.43 [2.00, 2.83]
**T1—WORRY** (median [IQR])	3.25 [3.00, 3.75]	3.25 [3.00, 4.00]	3.50 [3.06, 4.00]	3.25 [3.00, 4.00]
**T1—CONDWORK** (median [IQR])	2.43 [2.00, 3.07]	2.43 [2.00, 2.86]	2.57 [2.18, 2.86]	2.14 [1.71, 2.57]

Abbreviation: no., number; MSPSS, Multidimensional Scale of Perceived Social Support; COPE, Brief Cope; IQR, InterQuartile Range; Psych History, psychiatric history; CONDWORK, working conditions.

**Table 3:** Estimates (standard-errors) of the models for the DASS-21 subscales. Depression and anxiety scales were square root transformed.

Parameter	Depression	Anxiety	Stress
Intercept	−0.69(0.73)	−2.14(0.67) **	−10.96(4.05) **
Time (T1 vs. T0)	0.11(0.15)	−0.1(0.15)	1.68(0.96)
Age	0(0.01)	0(0.01)	−0.03(0.04)
Gender (Female vs. Male)	0.18(0.19)	0.19(0.18)	0.02(1.05)
Occupation (Ref = Physicians)			
Nurses	−0.1(0.21)	0.58(0.2) **	−0.84(1.22)
Clerks	−0.05(0.34)	0.2(0.31)	−0.53(1.92)
Other healthcare	−0.16(0.22)	0.22(0.21)	−0.14(1.26)
Ward COVID (Yes vs. No)	−0.11(0.14)	−0.1(0.14)	−1.6(0.85)
WORRY	0.39(0.09) ***	0.54(0.09) ***	2.41(0.53) ***
CONDWORK	0.51(0.09) ***	0.36(0.08) ***	4.59(0.5) ***
Psych history (Yes vs. No)	0.63(0.17) ***	0.64(0.16) ***	4.88(0.93) ***
Emergency training (Yes vs. No)	−0.44(0.2) *	−0.43(0.18) *	−2.42(1.07) *
MSPSS	−0.02(0.01) **	−0.003(0.005)	−0.05(0.03)
COPE—Problem-Focused	−0.47(0.16) **	−0.34(0.14) *	−2.64(0.85) **
COPE—Emotion-Focused	0.55(0.23) *	0.38(0.21)	3.98(1.24) **
COPE—Avoidant	1.03(0.19) ***	0.93(0.17) ***	5.88(1.02) ***
T1: Occupation = Nurses	0.33(0.21)	0.25(0.2)	1.5(1.29)
T1: Occupation = Clerks	0.56(0.34)	0.37(0.32)	2.78(2.1)
T1: Occupation = Other healthcare	0.19(0.22)	0.14(0.21)	0.06(1.35)

*** *p* < 0.0001; ** *p* < 0.01; * *p* < 0.05. Abbreviation: Ref, reference; CONDWORK, working conditions; Psych History, psychiatric history; MSPSS, Multidimensional Scale of Perceived Social Support, COPE, Brief Cope.

**Table 4:** Estimates (standard-errors) of the models for the MBI Emotional Exhaustion, ISI total score, IES-R Intrusion, and State Anger. Square root transformation was applied to the MBI Emotional Exhaustion scale, ISI total score, and IES-R Intrusion scale, while ordered quantile normalization was used for the State Anger scale.

Parameter	MBI EMO EX	ISI TOT	IES Intrusion	STATE Anger
Intercept	0.85(0.74)	0.44(0.28)	−1.01(0.19) ***	−2.26(0.44) ***
Time (T1 vs. T0)	0.07(0.13)	0.02(0.06)	−0.02(0.03)	0.3(0.09) **
Age	0.01(0.01)	0.01(0.003) ***	0.005(0.002) *	0.002(0.004)
Gender (Female vs. Male)	0.37(0.2)	0.08(0.08)	0.07(0.05)	−0.21(0.12)
Occupation (Ref = Physicians)				
Nurses	−0.32(0.21)	0.28(0.08) ***	0.16(0.06) **	0.06(0.13)
Clerks	−0.26(0.33)	0.12(0.13)	−0.03(0.09)	0.06(0.2)
Other healthcare	−0.52(0.22)	0.16(0.09)	−0.03(0.06)	0.23(0.13)
Ward COVID (Yes vs. No)	−0.1(0.13)	−0.1(0.05)	−0.02(0.03)	0.16(0.09)
WORRY	0.32(0.09) ***	0.19(0.04) ***	0.13(0.02) ***	0.23(0.06) ***
CONDWORK	0.55(0.08) ***	0.29(0.03) ***	0.15(0.02) ***	0.19(0.05) ***
Psych history (Yes vs. No)	0.74(0.18) ***	0.23(0.07) ***	0.17(0.05) ***	0.2(0.1)
Emergency training (Yes vs. No)	−0.35(0.2)	−0.22(0.08) **	−0.14(0.05) **	−0.15(0.12)
MSPSS	−0.02(0.01) **	−0.002(0.002)	−0.002(0.001)	−0.002(0.003)
COPE—Problem-Focused	−0.51(0.16) **	−0.09(0.06)	−0.02(0.04)	−0.22(0.09) *
COPE—Emotion-Focused	0.79(0.23) ***	0.07(0.09)	0.15(0.06)	0.34(0.14) *
COPE—Avoidant	0.84(0.19) ***	0.35(0.07) ***	0.37(0.05) ***	0.54(0.11) ***
T1: Occupation = Nurses	0.21(0.18)	0.09(0.08)	0.04(0.04)	0.0003(0.12)
T1: Occupation = Clerks	0.74(0.29) *	0.15(0.12)	0.09(0.07)	0.32(0.2)
T1: Occupation = Other healthcare	0.13(0.18)	0.003(0.08)	0.04(0.05)	−0.25(0.13)

*** *p* < 0.0001; ** *p* < 0.01; * *p* < 0.05. Abbreviation: Ref, reference; CONDWORK, working conditions; Psych History, psychiatric history; MSPSS, Multidimensional Scale of Perceived Social Support, COPE, Brief Cope; MBI Emo Ex, Maslach Burnout Inventory-Emotional Exhaustion; ISI TOT, Insomnia Severity Index Total score; IES Intrusion, Impact of Event Scale-Intrusion.

## Data Availability

Data cannot be shared because participants did not provide written informed consent for it.

## Supplementary Materials

The following supporting information can be downloaded at: https://www.mdpi.com/article/10.3390/jcm11092317/s1, Table S1: Descriptive statistics of investigated psychometric variables; Table S2: Observed ranges of investigated psychometric variables; Table S3: Estimates (standard-errors) of the models. Square root transformation was applied to MBI depersonalization scale, while scores on the MBI professional realization scale were raised to the power of two; Table S4: Estimates (standard-errors) of the models. Square root transformation was applied to scores on both IES-R Avoidance and Hyperarousal scales.
