# Supplementary material for: The “Healthcare Workers’ Wellbeing [Benessere Operatori]” Project: A Longitudinal Evaluation of Psychological Responses of Italian Healthcare Workers during the COVID-19 Pandemic
Source: J Clin Med. 2022 Apr 21;11(9):2317. doi: 10.3390/jcm11092317 (PMC9103992; doi:10.3390/jcm11092317)
Supplement: Supplementary file 1 [file jcm-11-02317-s001.zip › jcm-1661569-supplementary.pdf]

Table S1. Descriptive statistics of investigated psychometric variables.

	Physicians (n=91)		Nurses (n=97)		Clerks (n=22)		Other Healthcare (n=81)	
	**mean**	**sd**	**mean**	**sd**	**mean**	**sd**	**mean**	**sd**
T0 - DASS DEPR	8.79	8.64	9.51	8.94	11.91	10.93	8.3	7.88
T0 - DASS ANX	4.53	5.97	7.63	7.43	7.73	8.93	5.88	6.55
T0 - DASS STRESS	15.23	9.82	15.57	9.19	18.09	11.49	14.96	9.01
T0 - ISI TOT	7.45	3.46	9.48	4.18	9.68	4	8.4	3.92
T0 - IES INTR	0.83	0.8	1.23	0.87	1.14	0.95	0.86	0.85
T0 - IES AVOID	0.77	0.7	1.05	0.72	1	0.72	0.81	0.68
T0 - IES HYPER	0.92	0.81	1.16	0.89	1.27	0.98	1.03	0.86
T0 - STATE ANG	19.27	6.4	21.31	8.98	21.5	8.08	20.3	7.06
T0 - MBI EMO EX	20.22	12.63	20.34	14.46	21.32	11.21	17.31	13.08
T0 - MBI DEPERS	6.69	6.05	6.39	6.29	5.18	3.78	3.15	4.22
T0 - MBI PROF REAL	36.57	8.18	37.58	7.5	24.55	12.53	34.96	10.16
T1 - DASS DEPR	10.29	10.01	11.28	9.71	14.45	9.8	10	9.1
T1 - DASS ANX	4.79	6.93	8.06	7.12	8.45	8.14	6.12	6.78
T1 - DASS STRESS	16.97	10.62	17.73	9.99	20.64	10.86	16.15	10.17
T1 - ISI TOT	7.65	3.36	9.68	3.86	9.91	3.61	8.35	3.85
T1 - IES INTR	0.76	0.71	1.18	0.82	1.14	0.83	0.85	0.86
T1 - IES AVOID	0.65	0.6	1.04	0.72	0.95	0.73	0.81	0.63
T1 - IES HYPER	0.86	0.74	1.13	0.83	1.23	0.82	1.04	0.9
T1 - STATE ANG	21.35	7.95	22.86	9.6	25.95	10.6	21.54	9.62
T1 - MBI EMO EX	21.87	14.3	21.41	13.92	27.64	14.37	18.3	13.76
T1 - MBI DEPERS	7.58	7.05	6	5.93	6.59	6.33	3.56	4.81
T1 - MBI PROF REAL	35.47	8.73	36.05	8.63	24.18	13.22	34.84	10.15

Table S2. Observed ranges of investigated psychometric variables.

	Physicians (n=91)		Nurses (n=97)		Clerks (n=22)		Other Healthcare (n=81)	
	**min**	**max**	**min**	**max**	**min**	**max**	**min**	**max**
T0 - DASS DEPR	0	32	0	36	0	42	0	38
T0 - DASS ANX	0	26	0	30	0	36	0	30
T0 - DASS STRESS	0	42	2	40	0	42	0	42
T0 - ISI TOT	3	18	0	21	3	19	3	19
T0 - IES INTR	0	3.75	0	3.13	0	3.63	0	3.5
T0 - IES AVOID	0	3	0	3.38	0	2.5	0	2.88
T0 - IES HYPER	0	3.5	0	3.83	0	4	0	3.67
T0 - STATE ANG	15	57	15	53	15	41	15	57
T0 - MBI EMO EX	1	54	0	54	5	48	0	54
T0 - MBI DEPERS	0	24	0	24	0	12	0	20
T0 - MBI PROF REAL	15	48	16	48	4	47	3	48
T0 - WORRY	1.50	4.50	1.75	4.50	1.75	4.75	1.75	5
T0 - CONDWORK	1	4.5	1.2	5	1.6	5	1	4.17
T0 - MSPSS	33	84	12	84	31	84	27	84
T0 - COPE – Problem-Focused	1.25	4	1	4	1	3.75	1.25	4
T0 - COPE –_Emotion-Focused	1.25	3.25	1	3.33	1.25	2.83	1	3.42
T0 - COPE – Avoidant	1	2.5	1	2.88	1	3	1	2.75
T1 - DASS DEPR	0	42	0	38	0	30	0	36
T1 - DASS ANX	0	34	0	34	0	24	0	28
T1 - DASS STRESS	0	40	0	40	2	36	0	38
T1 - ISI TOT	3	16	2	21	3	17	3	18
T1 - IES INTR	0	3.63	0	3.38	0	2.50	0	3.38
T1 - IES AVOID	0	2.5	0	3	0	2.625	0	2.75
T1 - IES HYPER	0	3.17	0	3.33	0	2.8	0	3.50
T1 - STATE ANG	15	60	15	60	15	56	15	58
T1 - MBI EMO EX	0	52	0	54	3	50	0	52
T1 - MBI DEPERS	0	24	0	21	0	25	0	20
T1 - MBI PROF REAL	0	48	9	48	0	45	0	48
T1 - WORRY	1.75	5	1.5	5	1.5	4.5	1.5	5
T1 - CONDWORK	1	4.57	1.29	4.43	1.43	3.57	1	4

Table S3. Estimates (standard-errors) of the models. Square root transformation was applied to MBI depersonalization scale, while scores on the MBI personal accomplishment scale were raised to the power of two.

Parameter	MBI DEPERS	MBI PROF REAL
Intercept	2.66(0.67)***	422.9(286.12)
Time (T1 vs T0)	0.03(0.12)	-57.01(56.57)
AGE	-0.02(0.01)***	3.28(2.59)
GENDER (Female vs Male)	-0.32(0.18)	56.15(75.6)
Occupation (Ref= Physicians)		
Nurses	-0.33(0.19)	37.86(82.95)
Clerks	-0.42(0.3)	-610.84(130.64)***
Other healthcare	-0.95(0.2)***	-76.07(86.02)
WARD COVID (Yes vs No)	0(0.12)	25.89(54.26)
WORRY	0.19(0.08)*	-34.57(35.11)
CONDWORK	0.25(0.07)***	-10.82(32.54)
PSYCH HISTORY (Yes vs No)	0.26(0.16)	-157.58(67.14)*
EMERGENCY TRAINING (Yes vs No)	-0.03(0.18)	51.43(76.76)
MSPSS	-0.02(0.0049)***	3.09(2.11)***
COPE – Problem-Focused	-0.45(0.14)**	346.52(61.14)
COPE – Emotion-Focused	0.57(0.21)**	-115.34(89.52)
COPE – Avoidant	0.46(0.17)**	-2.2(73.26)
T1 : Occupation = Nurses	-0.05(0.17)	-26.92(75.76)
T1 : Occupation = Clerks	0.21(0.27)	53.41(123.13)
T1 : Occupation = Other healthcare	0.05(0.17)	54.68(78.8)

*** $p < 0.0001$; ** $p < 0.01$; * $p < 0.05$.

Table S4. Estimates (standard-errors) of the models. Square root transformation was applied to scores on both IES-R Avoidance and Hyperarousal scales.

Parameter	IES Avoidance	IES Hyperarousal
Intercept	-0.7(0.18)***	-0.77(0.19)***
Time (T1 vs T0)	-0.1(0.04)**	-0.08(0.04)*
AGE	0.003(0.002)*	0.004(0.002)*
GENDER (Female vs Male)	0.07(0.05)	0.07(0.05)
Occupation (Ref= Physicians)		
Nurses	0.1(0.05)	0.08(0.06)
Clerks	0(0.08)	0(0.09)
Other healthcare	-0.03(0.06)	0.01(0.06)
WARD COVID (Yes vs No)	-0.02(0.04)	-0.07(0.04)
WORRY	0.12(0.02)***	0.16(0.02)***
CONDWORK	0.07(0.02)**	0.14(0.02)***
PSYCH HISTORY (Yes vs No)	0.09(0.04)*	0.19(0.04)***
EMERGENCY TRAINING (Yes vs No)	-0.04(0.05)	-0.12(0.05)*
MSPSS	-0.002(0.001)	-0.003(0.001)
COPE – Problem-Focused	-0.04(0.04)	-0.06(0.04)
COPE – Emotion-Focused	0.11(0.06)*	0.15(0.06)*
COPE – Avoidant	0.46(0.05)***	0.32(0.05)***
T1 : Occupation = Nurses	0.09(0.05)	0.07(0.05)
T1 : Occupation = Clerks	0.08(0.08)	0.1(0.09)
T1 : Occupation = Other healthcare	0.1(0.05)	0.09(0.06)

*** $p < 0.0001$; ** $p < 0.01$; * $p < 0.05$.
